# Early autologous and/or allogeneic stem cell transplantation for adult patients with advanced stage T- lymphoblastic leukemia/lymphoma or Burkitt lymphoma. A retrospective single-centre analysis

**DOI:** 10.1007/s00277-024-05979-3

**Published:** 2024-09-03

**Authors:** N. Steiner, K. Baier, D. Ritter, J. Rudzki, G. Hetzenauer, S. Köck, B. Kircher, E. Gunsilius, D. Wolf, D. Nachbaur

**Affiliations:** University Hospital of Internal Medicine V Hematology and Oncology, Medical University of Innsbruck, Anichstrasse 35, 6020 Innsbruck, Austria

**Keywords:** T-ALL/LBL, BL, ASCT, AlloSCT

## Abstract

T-cell acute lymphoblastic leukemia/lymphoma (T-ALL/LBL) and Burkitt lymphoma (BL) are uncommon, highly aggressive diseases originating either from immature precursor T cells or from mature B cells in BL. We retrospectively analyzed the outcome of an early autologous and/or allogeneic stem cell transplantation (SCT) concept in 28 patients with advanced stage T-ALL/LBL and BL after three to four remission induction/consolidation chemotherapy cycles. Considering only patients in first complete remission (CR), the 5-year overall survival (OS) and event-free survival (EFS) was 91% in patients with BL and 73% in patients with T-ALL/LBL with a 5-year relapse incidence (RI) of 9% in patients with BL and 27% in patients with T-ALL/LBL. All relapsing patients finally succumbed to the disease (*n* = 10) or complications/toxicity after having received a salvage allogeneic transplant (*n* = 5). Despite the low patient number our retrospective single-centre analysis by incorporating an early intensive high-dose chemo-/radiotherapy strategy with either autologous or allogeneic stem cell transplantation, although preliminary, show promising long-term outcome. Further studies are highly warranted to better define those patients who might benefit most from such a treatment approach.

## Introduction

T lymphoblastic leukemia/lymphoma (T-ALL/LBL) and Burkitt lymphoma (BL) are uncommon, highly aggressive B and T cell neoplasms [1, 2]. T-ALL/LBL is committed to immature precursor T lymphoblasts, clinically characterized by bone marrow or blood involvement with or without primary involvement of the thymus and nodal or extranodal sites. It is more common in male adolescents with a male to female ratio of 2.5. A bone marrow involvement of > 25% blasts is used as the threshold for defining leukemia [2–7].

Burkitt lymphoma originates from mature B cells located in the germinal center or post germinal center and can be divided into three distinct entities (i) endemic, which is associated with the Epstein-Barr virus occurring mainly in equatorial Africa and South America and mainly affecting children under the age of 18 with an incidence of 3–6/100.000 per year (ii) immunodeficiency-related, typically associated with HIV with an incidence of 22/100.000 per year in the United States and (iii) sporadic BL, which occurs mainly in Europe, East Asia and North America, with a median age at diagnosis of 45 years and an incidence of 2.5/ per million per year in adults [1, 8–10]. The clinical presentation is characterized by an extremely short doubling time often presenting in extra nodal (e.g. abdominal) sites or as an acute leukemia (1,10). Translocations involving the *myc* oncogene on chromosome 8 are highly characteristic but not specific [11].

Current treatment approaches using ALL-based regimen with or without autologous stem cell transplantation or DA-EPOCH give survival rates of 90% for BL and 70% for adult T-ALL/LBL [12, 13].

Herein, we report our single-centre approach using a short and intensive ALL-like induction/consolidation according to the respective GMALL protocols followed by autologous and/or allogeneic SCT for the treatment of advanced T-ALL/LBL and BL.

## Patients and methods

### Patient demographics

Between Dec 2007 and May 2023, 28 patients with either advanced stage T-ALL/LBL (*n* = 13) or advanced BL (Ann Arbor III-IV, *n* = 15), received a first autologous (*n* = 22) or first allogeneic (*n* = 6) SCT whenever possible in first CR after 3–4 ALL-based induction/consolidation according to the GMALL protocol 07/2002 amendment 6 for T-ALL/LBL and according to the GMALL-B-ALL/NHL protocol 2002 Amend IX 03/2010 for patients with BL. Patient demographics are shown in Table 1. Five patients (39%) in the T-precursor cohort initially presented as T-ALL. Eight patients with T-ALL/LBL and 11 patients with BL achieved a first CR before stem cell transplantation. All patients gave written informed consent. All autologous stem cell grafts were analyzed by clonality testing by TCR or IG gene rearrangement, next generation flow cytometry, conventional cytogenetics, and *myc*-FISH analysis in patients with BL. Standard BEAM (*n* = 22) conditioning was used for autologous transplants. Patients with a very high-risk constellation according to the respective GMALL protocol were undergoing a first allogeneic transplant, either received mainly TBI-based MAC or BUFLU- or FBM-based RIC conditioning regimen.

**Table 1:** Characteristics of T-ALL/LBL and Burkitt’s NHL patients receiving a first autologous or allogeneic SCT

	T-ALL/LBL	Burkitt’s NHL	
	(*n*=13)	(*n*=15)	*p* value
Median age at diagnosis (years (range))	27 (19-68)	20 (41-65)	<0.0075
Male:female ratio	10:3	11:4	
Median time from diagnosis to first SCT (months (range))	3 (5-49)	4 (5-6)	n.s.
Remission induction			
GMALL 07-03 protocol			
Ind. I, II, Cons I	8	n.a.	
Other regimen	5	n.a.	
GMALL B-ALL/NHL 2002 protocol			
A1, B1, C1±A2	n.a.	10	
other regimen	n.a.	5	
Disease status at time of first SCT			
CR1	8	11	
not in CR1	5	4	
Type of first SCT			
autologous	9	13	
allogeneic	4	2	
HLA-identical sibling donor	1	1	
10/10 matched unrelated donor	3	1	
Conditioning for first SCT			
autologous			
BEAM	9	13	
allogeneic			
MAC, TBI (12 Gy)-containing	3	1	
MAC, chemotherapy alone	1	0	
RIC (BUFLU)	1	0	
Median CD34 cell number (x10^6^/kg BW, range)	5.21 (1.17-20.87)	13.74 (1.99-43.6)	n.s.
Median number of days to leukocyte engraftment (≥1.0 G/L)	18 (10-219)	11 (7-21)	n.s.

*Abbreviations: T-ALL/LBL, *T-cell acute lymphoblastic leukemia/lymphoma*; NHL, *non-Hodgkin Lymphoma*; SCT, *stem cell transplantation*; GMALL, *German multicenter study group for adult ALL*; Ind*, Induction therapy*; Cons*; consolidation*; B-ALL, *B-cell ALL*; CR, *complete remission*; HLA, *human leukocyte antigen*; BEAM, *carmustine, etoposide, cytarabine, melphalan*; MAC, *myeloablative conditioning*; TBI; *total body irradiation*; Gy, *Gray*; RIC, *reduced-intensity conditioning*; BUFLU, *busulfan, fludarabine*; BW, *body weight*; n.s., *not significant; *n.a*., not available

### Study endpoints

The primary endpoint was overall survival (OS). Secondary endpoints were event-free survival (EFS), non-relapse mortality (NRM), and relapse incidence (RI).

### Statistical methods

Data were retrospectively analyzed as of December 2022. All statistics were computed using NCSS Statistical Software version 19.0.5. The probabilities of OS were calculated using the Kaplan–Meier method from the date of the first transplant until death. EFS was calculated from the date of the first transplant until relapse or death whatever occurred first. The cumulative RI was calculated from the date of the first transplant until relapse with death without relapse as competing risk. The cumulative incidence of NRM was calculated from the date of the first transplant to the date of death with death from relapse as competing risk.

According to the EBMT statistical guidelines patients receiving a second allogeneic transplant because of relapse after first transplant were neglected and considered only once for calculations of OS, EFS, and NRM [14].

## Results

### Overall and event-free survival

The 5-year and 10-year OS of patients with BL was 67%. The OS of patients with T-ALL/LBL was 65 and 48% after 10 years.

**Fig. 1:**
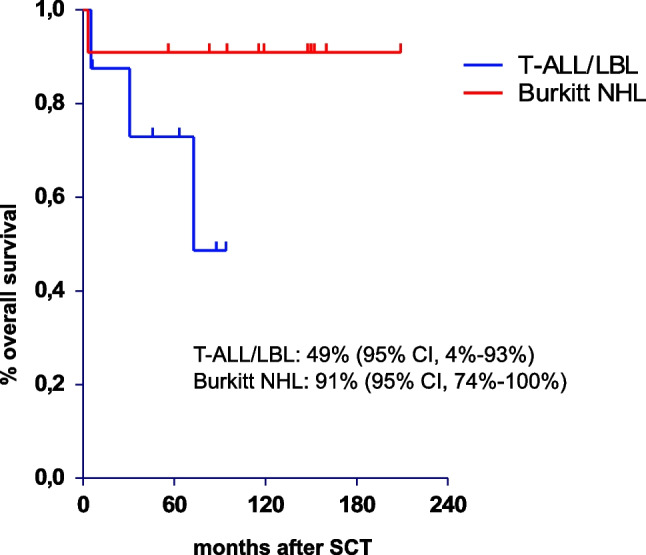
Overall survival of stem cell transplant patients with T-ALL/LBL and BL in CR1

In patients in first CR the 5-year and 10-year OS in patients with BL was 91%, and 73% and 49% in patients with T-ALL/LBL, respectively. (Fig. 1)**.**

**Fig. 2:**
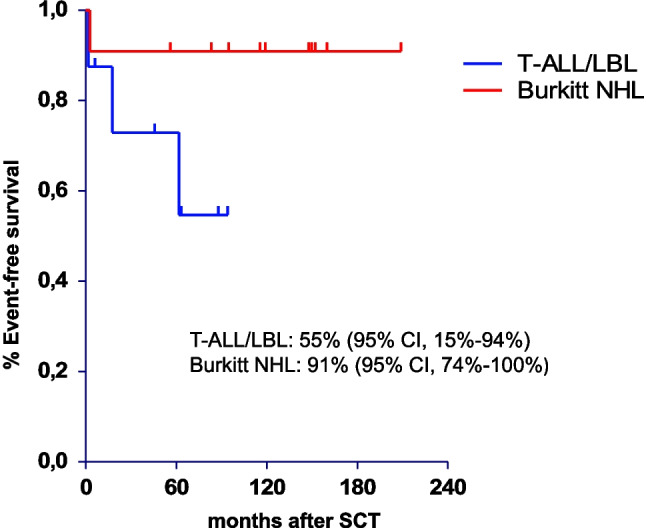
Event-free survival of stem cell transplant patients with T-ALL/LBL and BL in CR1

The 5- and 10-year EFS for patients with BL was constantly 67%. In patients with T-ALL/LBL the 5- and 10-year EFS was 65% and 52%. In patients in CR1, the 5- and 10-year EFS was 91% in patients with BL and 73% and 55% in patients with T-ALL/LBL (Fig. 2).

One patient suffered life-threatening multi-organ failure during induction phase 1 and was only able to receive an autologous SCT 20 months after diagnosis in partial remission 2.

### Non-relapse mortality and relapse incidence

Overall, 10/28 patients died, all of them having documented disease relapse after their first SCT. Of these, five pts. received a second allogeneic SCT either from a matched (*n* = 3) or mismatched unrelated/family donor (*n* = 2). Eight patients died either of disease relapse/progression and two from treatment related causes (one due to septicMOF with CT scan-documented disease progression on the day of death and another due to progressive multifocal JC virus-negative leukoencephalopathy). Of the remaining 18 patients without relapse after their first autologous (*n* = 13) or allogeneic (*n* = 4) transplant all were alive and in ongoing complete remission resulting in a non-relapse mortality after the first procedure of 0%.

**Fig. 3:**
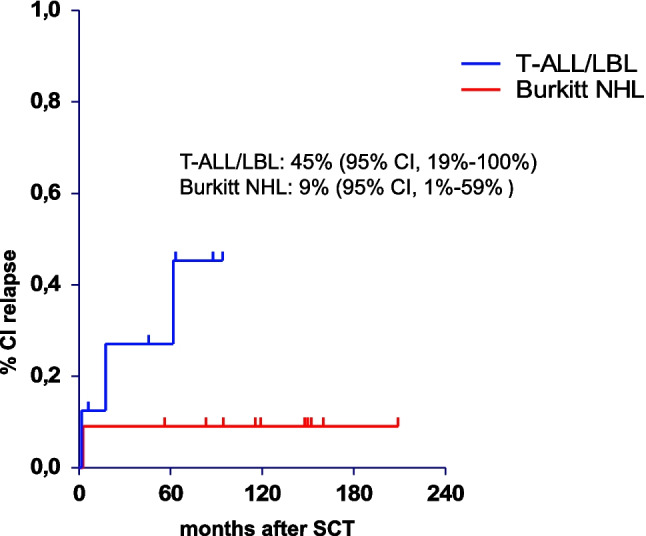
Cumulative incidence of relapse of stem cell transplant patients with T-ALL/LBL and BL in CR1

The incidence of relapse in patients with BL was 33% and 35% and 48% after five and ten years for patients with T-ALL/LBL. If only the patients in first CR were considered the overall RI was 9% for patients with BL and 45% for patients with T-ALL/LBL (Fig. 3).

## Discussion

This retrospective single-center analysis shows clear benefits of an early SCT approach to patients with advanced stage BL and T-ALL/LBL. It results in shorter treatment duration and surprisingly good tolerability of early intensification.

Nearly all patients underwent remission induction/consolidation according to the GMALL 07/2003 or the GMALL B-ALL/NHL 2002 protocol.

Patients with BL in first CR achieved a 10-year OS and EFS of 91%. Compared to a study analyzing the results of the GMALL B-ALL/NHL 2002 protocol in patients with BL, the 5-year OS was similar to our study, except for the longer duration of conventional treatment, namely 94% in patients < 55 years and 64% in patients > 55 years [15]. Similar results were found in a large study by Evens et al. of patients with BL treated at 30 different US cancer centers. The majority of these patients were treated according to the CODOX-M/IVAC regimen in combination with rituximab in 90% of patients [16]. This shows again that early SCT can achieve outcomes superior to those of previously used regimens, but with a dramatic reduction in treatment duration, namely a median time of four months from diagnosis to SCT. In addition, the improved treatment outcome is demonstrated when CR is achieved prior to SCT.

T-ALL/LBL patients in the first CR1 achieved a 5-year OS and EFS rate of identical 73%. In addition, the entire patient cohort reached a 5-year OS and EFS of 65%. Compared to a study published by Fredman et al. using the GMALL 07/2003 protocol in ALL and LBL in Israel in a cohort for 127 patients, a 5-year OS of 68% was achieved in the T-ALL group, similar to our study [17]. In an update of the GMALL study 08/2013, a 3-year OS rate of 78% was described in 208 T-ALL patients. In high-risk T-ALL/LBL patients in CR1 who received allogeneic SCT, a 3-year OS rate of 68% was observed with CIR and TRM rates of 26% and 15%, respectively [18].

In our study, we could demonstrate that comparable results can be achieved with a significant reduction in the median treatment duration of three months after diagnosis. The limitating factor in this comparison is the smaller number of patients enrolled.

A multicenter phase II study published in 2005 by the Dutch-Belgian Hemato-Oncology Cooperative Group (HOVON) analyzed the outcome of autologous SCT after short course of chemotherapy in patients with BL and LBL with a 5-year OS of 81% in patients with BL and 40% in patients with LBL. The reason for these rather unsatisfactory results could be the advanced stage of patients; namely 37% of patients with BL and 53% of patients with LBL belonged to the high-intermediate or high-risk group after aa-IPI [19]. In our study, we could nicely demonstrate that achieving prior CR is of more prognostic value than the advanced stage at the beginning of diagnosis. However, due to the small number of treated patients, every firm conclusion will remain limited in its full strength.

In addition, a meta-analysis by Hoelzer et al. of early autologous and allogeneic SCT for T-ALL/LBL showed that patients who received autologous SCT in CR1 achieved a DFS of 61%. However, patients who did not undergo autologous SCT in CR had a shorter DFS of only 47% [13]. This again indicates the clear benefit of SCT in CR1.

Furthermore, all patients in our study had an NRM rate of zero. This can be attributed to the short treatment duration with early SCT and the good tolerability of SCT. It highlights the value of implementing SCT and an intensified protocol from the very far beginning for a limited time in the treatment approach of BL and T-ALL/LBL patients to omit long-lasting chemotherapeutic-based regimens.

Nevertheless, our study has several limitations, such as limited patient number, single center analysis, retrospective nature of the study with missing data, and lack of MRD status including unestablished modalities and experience with assessing MRD years ago. All these factors may influence the analyses.

In conclusion, the results of our single-center study with a long overall observation period of 15 years stresses a clear benefit of early SCT in patients with advanced-stage BL and T-LBL in CR1 without major toxicity/mortality rates. Further research is necessary to better define those patients who might benefit most from such an approach incorporating better molecular subtyping, early MRD and PET diagnostics and to identify those patients who are at high risk of early relapse or with refractory disease requiring front-line allogeneic SCT.

## Data Availability

No datasets were generated or analysed during the current study.
